# A guide to human in vivo microcirculatory flow image analysis

**DOI:** 10.1186/s13054-016-1213-9

**Published:** 2016-02-10

**Authors:** Michael J. Massey, Nathan I. Shapiro

**Affiliations:** 1 Department of Emergency Medicine, Beth Israel Deaconess Medical Center, Harvard Medical School, 330 Brookline Avenue, Boston, MA 02215 USA; 2 The Center for Vascular Biology Research, Beth Israel Deaconess Medical Center, 99 Brookline Ave., Boston, MA 02215 USA

## Abstract

Various noninvasive microscopic camera technologies have been used to visualize the sublingual microcirculation in patients. We describe a comprehensive approach to bedside in vivo sublingual microcirculation video image capture and analysis techniques in the human clinical setting. We present a user perspective and guide suitable for clinical researchers and developers interested in the capture and analysis of sublingual microcirculatory flow videos. We review basic differences in the cameras, optics, light sources, operation, and digital image capture. We describe common techniques for image acquisition and discuss aspects of video data management, including data transfer, metadata, and database design and utilization to facilitate the image analysis pipeline. We outline image analysis techniques and reporting including video preprocessing and image quality evaluation. Finally, we propose a framework for future directions in the field of microcirculatory flow videomicroscopy acquisition and analysis. Although automated scoring systems have not been sufficiently robust for widespread clinical or research use to date, we discuss promising innovations that are driving new development.

**Electronic supplementary material:**

The online version of this article (doi:10.1186/s13054-016-1213-9) contains supplementary material, which is available to authorized users.

## Background

Section 1 introduces the various noninvasive microscopic imaging technologies that have been used to visualize the sublingual microcirculation in patients. We review basic differences in the cameras, optics, and light sources, as well as digital image capture. Section 2 describes techniques for image acquisition. In Section 3, we discuss aspects of video data management, including data transfer, metadata, and database design and utilization to facilitate the image analysis pipeline. Section 4 is dedicated to image analysis techniques and reporting. Section 5 discusses future directions and conclusions.

Orthogonal polarization spectral (OPS) imaging [1] and its successors, sidestream dark-field (SDF) imaging and CytoCam (Braedius Medical B.V., Huizen, the Netherlands) incident dark-field (IDF) imaging, are techniques that allow visualization of the sublingual microcirculation at the bedside. These techniques have been used in a number of studies involving the emergency department (ED) and intensive care (ICU) settings [2–41] and have broadly defined the importance of the microcirculation in pathophysiology and survival in critical illness. Prior efforts, including a consensus statement from a roundtable summit [7], have provided the foundation for approaches to image acquisition and analysis. Here, we seek to extend prior work and to provide a comprehensive guide to image acquisition, processing, and analysis based on a combination of the medical literature and our real-world experience with the goal of enabling an end user to implement similar approaches in their research and/or clinical settings. This guide targets the clinician, the clinician-researcher, and the technical user.

### Vessel classification

The microcirculation consists of a branching network of arterioles, capillaries, and venules. Large arterioles and venules have lumen diameters less than approximately 100 μm. Small vessels, composed of capillaries and postcapillary venules, are the primary sites of oxygen exchange between blood and tissue and have lumens with diameters that range between 0 and 20 μm. Medium-size vessels (precapillary arterioles and postcapillary venules) have lumen diameters between approximately 20 and 50 μm. Several earlier studies have shown that the flow in and the density of perfused small vessels (<20 μm) are altered in severe sepsis and that the observed small vessel dysfunction is associated with poorer outcomes [42–44]. Based on the physiologic importance to oxygen exchange, analysis of microvascular flow typically focuses on the 0–20 μm vessels.

## Section 1: available imaging technologies

### Cameras

The orthogonal polarization spectral dark-field (OPS-DF) imaging device (Cytometrics, Philadelphia, PA, USA) uses a high-intensity green-filtered light source that is linearly polarized to illuminate the tissue. Polarized reflected light is blocked by an orthogonally polarized analyzer while depolarized scattered light passes through to the camera. The OPS-DF device includes a dark-field optical element consisting of a filter with a black spot in the middle. OPS-DF has been phased out in favor of several technological innovations based on the ideas described as SDF imaging [20] or IDF illumination microscopy [45]. Several SDF/IDF cameras are commercially available for visualizing microcirculation in vivo. Table 1 compares technical specifications between commercial SDF/IDF devices.

**Table 1:** Comparison between SDF/IDF technical specifications

	Microscan (Microvision Medical B.V., Amsterdam, the Netherlands)		Capiscope HVCS (KK Technology, Honiton, UK)	Capiscope HVCS-HR^a^ (KK Technology)	Cytocam (Braedius Medical B.V., Huizen, the Netherlands)
Type	SDF		SDF	SDF	IDF
Image size (pixels)	720 × 480 (NTSC)	720 × 576 (PAL)	752 × 480	1280 × 1024	2208 × 1648
Resolution (μm/pixel)	1.45 (horizontal), 1.58 (vertical)^b^		0.92	0.81	0.66^c^
Field of view (μm)	1044 × 758 (NTSC)		692 × 442	1037 × 829	1457 × 1061
Frame rate (fps)	30 (NTSC)	25 (PAL)	Up to 87^d^	25^d^	25
Illumination time (ms)	10		0.5–2^d^	0.5–2^d^	2

^a^Capiscope HVCS-HR uses the same camera, illumination, and optics as the Capiscope HVCS with a modified sensor and electronics

^b^Measured using an NTSC version and Canopus ADVC110 video digitizer

^c^Measured using a 150 line-pairs per inch Ronchi ruling (Edmund Optics, Barrington, NJ, USA)

^d^Private communication with manufacturer

*IDF* incident dark field, *NTSC* national television system committee, *PAL* phase altering line, *SDF* sidestream dark field

The Microscan (Microvision Medical B.V., Amsterdam, the Netherlands) was the first commercial SDF device available and has been in use since 2007 [46]. It uses a progressive charge-coupled detector (CCD) camera with standard American (NTSC, 480 lines @ 30 Hz) or European (PAL, 576 lines @ 25 Hz) format analog video output. The optical path is isolated from a ring of six green light-emitting diodes (LEDs) located at the end of the probe. A disposable, transparent sterile cap covers the entire probe shaft. The NTSC version outputs 480 video lines at 30 Hz (30 frames per second). Analog video is converted to a computer-readable digital data stream via an analog-to-digital converter such as the Canopus ADVC-110 (Grass Valley, Montreal, QC, Canada). The type of video codec used is extremely important for image quality. The Canopus ADVC-110 uses a digital video (DV) codec, which outputs an image with dimensions 720 pixels × 480 pixels, a pixel aspect ratio of 1.1:1 (width:height), and a horizontal/vertical resolution of 1.45/1.58 μm/pixel. The field of view is approximately 1044 μm × 758 μm. The DV codec uses 4:1:1 (luma:chroma:chroma) digital sampling, such that all of the luminance but only one-quarter of each of the color channels are preserved, which is sufficient for the monochromatic camera in the Microscan. Each frame of a DV video is compressed using joint photographic experts group (JPEG) compression. DV or motion joint photographic experts group (M-JPEG) compressed video maintains image integrity sufficient for SDF or IDF image analysis. However, video capture devices that use moving pictures expert group-4 video codec (MP4) compression are not recommended because part of the moving pictures expert group (MPEG) compression algorithm samples image blocks from adjacent frames to form a representation of a subset of the video frames; thus, accurate estimation of blood flow is compromised because the integrity of individual frames is not preserved. To date, the Microscan is perhaps the most widely utilized device and has the largest publication track record.

The CapiScope HVCS (KK Technology, Honiton, UK) is an SDF device with a DV camera that connects to a computer via a USB port. The CapiScope HVCS has similar image size as the Microscan device (752 pixels × 480 pixels) with an aspect ratio of 1:1, a resolution of 0.92 μm/pixel, and a field of view of 692 μm × 442 μm. The newer HVCS-HR version has an image size of 1280 pixels × 1024 pixels, with a resolution of 0.81 μm/pixel and a field of view of 1037 μm × 829 μm. A low-power lens is available to reduce the magnification by approximately a factor of two and increase the field of view likewise. The CapiScope connects to a laptop computer running the CapiScope software via a USB 2.0 connection. Output video is uncompressed. The CapiScope has a fixed focal plane located a few millimeters below the probe tip. Focus is achieved by a focus ring that mechanically advances and retracts a disposable probe cover. LED intensity is controlled programmatically. The CapiScope HVCS requires a proprietary video capture and analysis package. It also is compatible and has been combined with GlycoCheck (Microvascular Health Solutions Inc., Salt Lake City, UT, USA), a software package that measures and reports the blood perfused boundary region among other parameters which are proposed to assess glycocalyx thickness. While previous studies have primarily used Microscan images with GlycoCheck software analysis, compatibility with GlycoCheck is an attractive feature of the CapiScope HVCS.

The CytoCam (Braedius Medical B.V.) is a high-resolution IDF DV camera illuminated with 12 high-intensity, short-pulsed LEDs. It includes a specially designed acromat multiplet objective lens and a high pixel-density image sensor synchronized with a very short duration (2 ms) LED illumination. The camera outputs uncompressed digital image sequences with image size 2208 pixels × 1648 pixels, a pixel aspect ratio of 1:1, resolution 0.66 μm/pixel, and a 1457 μm × 1061 μm field of view operating at 25 Hz (25 fps). The CytoCam imaging head is tethered to a computer via a cable and specialized PCIe interface card. Focus is actuated by an internal stepping motor that is controlled programmatically. The operator adjusts focus and LED intensity through the CytoCam Tools user interface (UI). The CytoCam includes a proprietary video capture and playback software package, CytoCam Tools. CytoCam Tools is a capture and analysis suite, designed exclusively for the CytoCam IDF device (Braedius Medical B.V.). It includes a UI for controlling the CytoCam via a desktop or all-in-one computer. The user enters patient and study information in specified fields of a dialog box. The user then navigates between controls for brightness and focus and to capture a video sequence. CytoCam Tools has a modular design that allows the user to add modules for video editing, stabilization, and analysis. The superior resolution and fast strobe makes the CytoCam an attractive choice.

Recent methodological studies comparing SDF imaging and CytoCam IDF imaging in healthy subjects [40] and neonates [35] observed 20–30 % more capillaries using CytoCam IDF than when using SDF in the same subjects. These differences may be due to a combination of: the IDF light source design that directs the illumination toward the optical axis (see figure in [35]); an enhanced depth of focus and resolution because of the greater sensor pixel density; and a sharper multi-element objective lens. These results suggest that measurements of microcirculatory small vessel density may differ depending on the camera system used. However, additional assessments are needed before a final conclusion is reached.

## Section 2: image capture and quality

Intrinsic image quality depends on the optical resolution, depth of focus, aberrations, sensor noise, and illumination properties of a camera system. Subsequent image processing may also affect image quality. Good image capture technique is an essential component for microcirculatory flow analysis. We have previously proposed a set of objective criteria for evaluating image acquisition quality that may be used to select images appropriate for analysis [21]. The components of the image quality criteria are illumination, duration, focus, content, stability, and pressure, as outlined in the following.

### Image quality

#### Illumination

Mucosal tissue that is imaged by SDF/IDF devices typically has low contrast. In our experience the required dynamic range encompasses about 6–7 bits. Spikes in apparent brightness appear as artifacts where specular reflections are imaged, such as along saliva bubble boundaries, or along mucosal surfaces that are not in good contact with the flat probe tip. In low illumination, camera sensor response is nonlinear and the signal-to-noise ratio is worse than in average or high illumination. On the other hand, the risk of extremely high illumination is that the signal in oversaturated pixels cannot be recovered. To maximize the signal-to-noise ratio, we recommend that illumination is maximized such that the peak of the image histogram lies well above the 50 % gray level, even if the darkest image pixels are still gray.

#### Focus

Good focus on the small vessels in the field of view is an important adjustment for optimal image quality. It is essential for accurate analysis of vessel diameters, blood flow rates, and estimates of capillary density. Furthermore, out of focus vessels lose contrast and are less visible. Higher-resolution cameras with higher pixel density (pixels/μm) sensors have greater depth of field. The CytoCam has more depth of field than the Microscan.

#### Duration

A captured image sequence should span sufficient duration such that stability and/or pressure artifacts can be adequately determined. The roundtable consensus guideline calls for 10 seconds of continuous, steady capture [7]. More recent reports recommend a duration of 3–5 seconds for accurate flow and density measurements [21].

#### Content

Content is a broad image quality category that encompasses vessel architecture, saliva, blood, and bubbles. We recommend that users find an optimal sublingual vascular bed, with a mix of medium and small vessels in the plane of focus that are not looped. Sublingual vascular beds vary by patient. Sublingual mucosa is composed of loose connective tissue with a three-dimensional microvascular structure. Arterioles tend to lie further below the sublingual mucosal surface than venules. As the mucosal tissue approaches connection points or surfaces, the vasculature forms arrays of repeating, looped arterioles. These looped structures usually lie far enough away from postcapillary venules that the venules are out of focus or outside the field of view. Images with a majority of looped vessels are traditionally excluded from analysis. A film of bubbles or contaminated saliva between the probe tip and the mucosa will occlude the optical path and promote specular or diffuse light scattering outside the tissue of interest. Saliva or bubbles that become adhered to the probe tip can be removed by gently dragging the probe tip along the tongue. Bubbles that appear along one side of the image usually indicate that the probe tip is tilted slightly away from flush with the surface. In that case, altering the probe or patient position may be more effective than forcing good contact with the mucosa.

#### Stability

Stability and pressure artifacts become apparent when observing live video or in captured sequences. Stability or motion artifacts arise when the camera is moving or when the tissue is moved relative to the camera. Tissue movement may be due to forces applied by the clinician or because the patient is moving their tongue. The clinician can stabilize the camera by resting their hands on the patient’s face. One technique is to have the patient roll their tongue up or to the side. Often the patient cannot hold their tongue still. Sometimes, a patient can relax their tongue over the probe and close their mouth slightly to obtain a good scan. For intubated patients, a tongue depressor can also be used to help open the mouth and raise the tongue for access. Shorter pulsed illumination sources can mitigate some of the risks of image blurring due to a shaky camera as well as blurring of high-velocity red blood cells (RBCs). See Table 1 for a comparison of illumination pulse durations of current SDF and IDF cameras.

#### Pressure

In our experience, this is perhaps the most important artifact and provides the greatest threat to analysis validity. Pressure artifacts which occur when the user applies too much pressure on the tissue can lead to a mechanical occlusion of flow. A pressure artifact will be manifested by small vessels that either drop out or appear to have disturbed microcirculatory flow along with particular flow disruptions in medium and/or large vessels. Larger thin-walled venules can serve as an indicator of iatrogenic pressure, because they are mechanically more sensitive to pressure and should occlude first. Technical solutions to this problem have been proposed. Balestra et al. [47] have demonstrated a vacuum-based image acquisition stabilizer that has the dual effect of stabilizing the camera on the mucosal surface and reducing pressure artifacts; however, this device has not made its way into routine use.

Prior to analysis, captured videos may be selected based on an image quality score (IQS). Videos are rejected if they fail the quality test in any of the categories. Researchers have discussed various methods to mitigate potential image artifacts [7,36,48]. Like ultrasound, sublingual SDF/IDF imaging requires substantial operator training to obtain best-quality images on a spectrum of patients and illness. Operator training may include some suggested best practices to optimize operator comfort and usability.

### Hands-free operation

Scanning operations are controlled by a “base” computer connected to, but away from, the camera for all SDF/IDF cameras. This presents a logistical challenge to hold the camera steady and in the right position while simultaneously operating a computer-based key. To address this, we have implemented foot switch controls for hands-free operation on several different SDF/IDF cameras. Off-the-shelf foot pedal controls can be programmed either through software or firmware. Software-controlled foot pedals require a software driver running on the device machine. Firmware-based controls are programmed once to operate on any system. The switch emulates any combination of keyboard or mouse clicks. Thus, any camera control application that has keyboard shortcuts will support these foot pedals. For example, on the CytoCam, we utilize a firmware programmable three-pedal foot switch (Savant Elite; Kinesis, Bothell, WA, USA). The pedals are mapped to keyboard shortcuts specified by the SDF/IDF manufacturer to step through the UI and to start/stop scanning.

## Section 3: data

### Data transmission

SDF/IDF data analysis is cumbersome because of the large video data files and steep analysis learning curve. Like others, we have opted for a centralized solution for multicenter studies. Three commonly implemented data delivery options are commercial cloud-based storage, customized data transfer software and servers, and manual data transfer through physical media.

### Data selection

As already described, we use an IQS that is a composite assessment of six categories: illumination, duration, focus, content, stability, and pressure. Videos are assigned a score of 0 = good, 1 = acceptable, or 10 = unacceptable for each category. The composite IQS is the sum of the scores from all categories. Any video with IQS ≥10 is discarded from future analysis. Videos can be sorted by the IQS at each timepoint and selected for further analysis. Some type of selection process, with a particular method of removing videos containing flow-occluding pressure artifacts, is essential for analyses with good fidelity.

### Automated data selection

Software algorithms have been developed that are specifically tuned to detect image quality deficiencies. There are a number of available solutions; however, our experience is that the software solutions for automated selection are still evolving. In our hands, we used a hybrid selection process with manual review to ensure inclusion of all components of a high-quality video.

### Workflow management

**Fig. 1:**
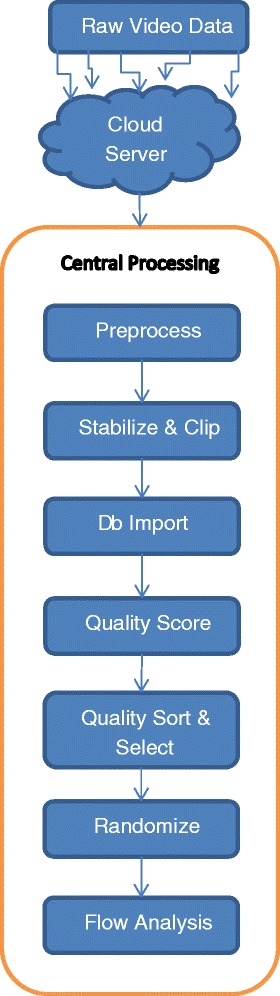
SDF video analysis workflow chart. Raw video is captured by multiple study sites into cloud storage folders. A central processing facility syncs with the cloud storage and moves files to an onsite server for further processing. Videos are preprocessed to remove noise, resize, enhance contrast, and correct background illumination inhomogeneity. Videos are stabilized and clipped to specified duration and dimensions for analysis. Clips that cannot be stabilized over a minimum duration are discarded. Files may be organized by study folders. Clipped and stabilized videos are reviewed for image quality and assigned an image quality score. A quality sorting algorithm can be applied to select files for randomization and further analysis. *Db* database

**Fig. 2:**
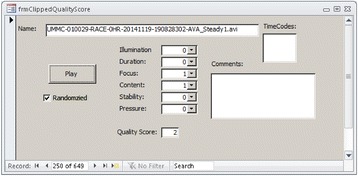
An IQS form. File name includes metadata showing, for example, study site, patient ID, study name, date, timestamp, calibration, and preprocessing. Clicking the “Play” button opens the video file in a video player. The “Randomized” checkbox is not editable and indicates that the video has been assigned a random ID and selected for analysis. A quality score (0 = good, 1 = acceptable, or 10 = unacceptable) is assigned for each image quality category. Fields are provided to record specific time codes and comments
- *Quality sort*. Usually a study will call for a fixed number of video data files to be analyzed at each timepoint for each patient. After all files for a patient and timepoint have been quality scored, a query can be implemented to sort by net quality score. Videos can be selected by their quality score up to the total number required by the study minus the number that have already been de-identified and added to the analysis queue.

An analysis workflow is represented schematically in Fig. 1 and can be summarized as follows:

- *Import*. Parse raw video filenames to extract metadata such as the camera serial number, the name of the study, the assigned patient number or ID, the study timepoint, the operator’s name, the scan number, the scan timestamp, etc. The specific type and variety of metadata may depend on the manufacturer’s output file format or available metadata, although we do recommend a process where this information is embedded in the video filename.
- *Preprocessing*. If preprocessed video files are imported, additional metadata should be provided including the specific preprocessing steps that were applied. Preprocessed video filenames can be constructed with human-readable annotations that can then be parsed for database import. Preprocessing may include contrast stretching, background correction, video stabilization, and automated clipping.
- *Quality scoring interface*. Figure 2 illustrates a sample UI that can be constructed within the database to facilitate compilation of the IQS. The UI can launch a video player to play the current video to be quality scored. The UI can have fields for the user to input a score for each category of image quality. In addition, the UI can have a field for any comments. Database filters can be applied to restrict review to unscored videos only. The IQS for each category and a net score can be stored in a table indexed by video name or unique ID.

## Section 4: image analysis

### Automated Vascular Analysis software

Automated Vascular Analysis (AVA 3.1; Microvision Medical B.V.) is an analysis software designed for the Microscan. It provides a semiautomated pipeline for preprocessing, video stabilization, vessel tracing, flow scoring, and computation of analytical parameters. AVA 3.1 generates a human-readable data file, called a Logbook, for each analyzed video file. The Logbook file contains several fields of metadata to describe the AVA 3.1 settings, calibration, video parameters, etc. Our group’s assessment of the accuracy of the automatic detection algorithm from now outdated versions was that it was not accurate enough for reliable image analysis. Thus, we used a semiautomated hybrid method (see later) where we manually identify the centerline and width of the vessels, and score the speed of flow, but then use the AVA 3.1 software to summate the various vessels by size and speed. Microvision has a new software release for which we have not assessed the accuracy of the automated scoring, but does contained improved algorithms.

### Preprocessing

Preprocessing is the first stage in any analysis pipeline. Contrast enhancement and background subtraction are the desired outcome of preprocessing. OPS/SDF/IDF images often suffer from low contrast and slowly varying background brightness due to high scattering and absorption inhomogeneity of the incident illumination by the underlying tissue.

### Stabilization and clipping

Image registration and stabilization are active areas of research in computer vision. It is beyond the scope of this document to review the applicability of various stabilization algorithms to SDF/IDF images. A basic principle of modern stabilization is to locate robust image features that can be tracked through a time sequence. For microcirculation images, these features may be the vessel points with the largest cross-sectional gradient [49] or at vessel bifurcations. Geometric transformation of the feature points can be modeled to two-dimensional camera motion. In brief, some preprocessing algorithms may be applied to improve subsequent vessel detection.

### Automated detection efforts

Manual and semiautomated microvascular analysis may ultimately be too time consuming to be practical in the clinical setting. Several researchers have reported promising efforts to automatically detect microvasculature in SDF/IDF images and determine perfusion parameters. However, the utility of these methods remains undefined. Demir et al. [49–51] reported an automated method for vessel segmentation and flow estimation using gradient-based preprocessing and statistical pixel intensity fluctuations. For the CytoCam (Braedius Medical B.V.), the manufacturer has developed automated vessel detection capabilities in the CytoCamTools analysis module. Preprocessing, vessel segmentation, and automated flow estimation in AVA 3.1 (Microvision Medical B.V.) were originally developed and coded by Dobbe et al. [12]. Bezemer et al. [4] developed algorithms to improve microvascular density assessment over semiautomated methods available in AVA 3.1 software by applying a thresholding scheme based on vessel centerline contrast. They quantified RBC flow as the standard deviation of pixel intensities in a number of frames normalized to the mean background intensity in a fixed pixel area. This method was able to discriminate between perfused and nonperfused microvasculature [4]. You et al. reported automated vessel centerline detection using a principal curve tracing algorithm [52] and a novel method for improved blood flow estimation from space–time images [53]. This is an evolving field that will be critical to the adoption of devices that assess sublingual microcirculatory flow.

### Calibration

Image data must be calibrated for accurate flow and density analysis. Usually it is sufficient to measure the pixel pitch using a Ronchi ruling oriented along the horizontal and vertical pixel directions. Microvision ships a calibration tool with the Microscan camera and the AVA 3.1 application contains an automated calibration module. CytoCam calibration has been hard-coded into the CytoCamTools application.

In AVA 3.1, calibration parameters (pitch) are stored in a settings file that is loaded by the user on startup. The settings file also contains the path(s) to the working study folder where de-identified files and analysis files are stored.

### Vessel segmentation

In our methodology, in the first stage of analysis, vessels are segmented from the background tissue by identifying the vessel centerline and lumen boundary using automated methods with manual verification and/or are manually drawn by an image analyst in AVA 3.1. When using the microscan, this is performed directly. If video capture is conducted using the CytoCam, then the videos are first rescaled and converted to avi format to fit into the AVA 3.1 workspace. Care must be taken to calibrate the rescaled image. Accurate image scale calibration facilitates accurate measurement of vessel dimensions and ultimately affects vessel size classification and measured vessel density. Working with an average image from a stabilized sequence, the user will draw vessel centerlines and lumens. A pen tablet can be used to facilitate accurate drawing. Creating an average image fills in plasma gaps and smoothes out noise. The user can select a range of frames to average. Usually 16–32 frames are sufficient to fill in plasma gaps and provide a good signal-to-noise ratio. If the image has good focus and illumination, some vessels can be easily detected in the average image using the AVA 3.1 automated vessel detection feature. A hybrid technique may be adopted where the automated detection is first used and then revised manually. In our experience, we often skip automated detection and draw all vessels by hand, finding this to be the most effective approach. Manual vessel tracing is very labor intensive. It can take up to 1 hour to trace and velocity score one video.

### Semiquantitative flow

After tracing, each vessel segment is scored for blood flow using a semiquantitative score. AVA 3.1 provides a UI such that the user can click on a vessel segment and assign a flow score to the segment while playing the stabilized video in a loop. The semiquantitative flow values are 0 = no flow, 1 = intermittent flow, 2 = sluggish flow, and 3 = continuous flow. In the perfusion calculations, any vessel segment with a flow score ≥2 (sluggish or greater) is perfused. We have found the approach of drawing vessels by hand and scoring by eye, and then using the AVA 3.1 software to calculate the various parameters, to be our group’s preferred approach. A detailed protocol for exporting AVA 3.1 Report files to produce each of the parameters, with accompanying routines, may be found in Additional file 1, which contains a Visual Basic script that can be executed in Microsoft Access to parse an AVA 3.1 Report file for analysis results. The data are inserted into a Microsoft Access table.

Semiquantitative flow estimation criteria were historically designed to evaluate the unique disturbed flow patterns in sepsis patients and therefore do not correlate proportionally with velocity. Additional file 2 presents a summary of semiquantitative flow characterization. To date, the most accurate method for estimating RBC velocity is through the use of space–time diagram analysis; however, this process for an individual vessel is quite time consuming and performing the measurement on all vessels in a given video is extremely time prohibitive such that we have found it not feasible for routine use [15].

### Microcirculatory parameters

A consensus of microcirculation parameters was initially proposed by a roundtable report [7]. Parameters were designed to facilitate automated and visual analysis methodologies while providing physiological metrics grounded in current clinical understanding of pathologies. In the literature, some parameter names have conflicting definitions and units. Some authors use different names for similar parameters. Table 2 summarizes the derived microcirculatory parameters and their references. The microvascular flow index (MFI) is a semiquantitative measure of perfusion quality, the De Backer score approximates vessel density using a line-crossing method, and the total vessel density (TVD) is another measure of the vessel density. Perfused vessel density (PVD) corresponds to a functional vessel density; that is, the density of functionally flowing capillaries. The proportion of perfused vessels (PPV) is the lineal proportion of flowing vessels.

**Table 2:** Microcirculation parameters

Microcirculation parameter	Information provided	Symbol/equation	Units	Measurement	References
Microvascular flow index	Perfusion quality (for small, medium, and large vessels^a^)	MFI	Arbitrary	The image is divided into four quadrants; a number is assigned for each quadrant according to the predominant type of flow (0 = no flow; 1 = intermittent; 2 = sluggish; 3 = continuous). The MFI results from the averaged values	[7, 14, 41, 54]
De Backer score	Vessel density	$\mathrm{V}{{\mathrm{D}}_{De}}_{Bac \ker}\kern0.5em =\kern0.5em Nx/L\mathit{\mathsf{g}}$	1/mm	The image is divided by three vertical and three horizontal lines; the De Backer score is calculated as the number of vessels crossing the lines divided by the total length of the lines	[7, 14, 41–43]
Total vessel density	Vessel density (for small, medium, and large vessels^a^)	TVD = *L*_*v*_/*A*_*FOV*_	mm/mm^2^	Total length of vessels is divided by the total surface of the analyzed area	[14, 41]
Proportion of perfused vessels (by length)	Perfusion quality (for small, medium, and large vessels^a^)	PPV_1_ = *L*_*p*_/*L*_*v*_	Percent	100 × number of perfused vessels is divided by the total number of vessels	[14, 41, 43]
Proportion of perfused vessels		PPV = *N*_*p*_/*N*_*v*_	Percent	100 × length of perfused vessels divided by total length of vessels	[7]
Flow heterogeneity index	Perfusion heterogeneity	*FHI* = (*MFI*_*max*_ − M*FI*_*min*_)/*MFI*_*avg*_	Unitless	The difference between the highest MFI and the lowest MFI is divided by the mean MFI. MFI is intended as the averaged MFI of each site	[14, 41]
Perfused vessel density		PVD = PPV * VD_De Backer_	1/mm	Vessel density × proportion of perfused vessels	[41]
Perfused vessel density	Functional vessel density (for small, medium, and large vessels^a^)	$$ \begin{array}{l}\mathrm{P}\mathrm{V}\mathrm{D}\kern0.5em =\kern0.5em {L}_p/{A}_{FOV}\kern0.5em \mathrm{or}\kern0.5em \\ {}\mathrm{P}\mathrm{V}\mathrm{D}\kern0.5em =\kern0.5em \mathrm{P}\mathrm{P}{\mathrm{V}}_1\kern0.5em *\kern0.5em \mathrm{T}\mathrm{V}\mathrm{D}\end{array} $$	mm/mm^2^	Total length of perfused vessels (sluggish or continuous) is divided by the total surface of the analyzed area (%)	[14, 36]

Three or five sites are evaluated. Parameters are usually stratified by vessel size

^a^Vessel diameter classification: <20 μm = small, 20–50 μm = medium, 50–100 μm = large

Modified from original by Donati et al. [13]

There are several variants of MFI in the literature, and as investigated by Pozo et al. [54] the MFI by quadrant is the most common and is assessed visually in a stabilized video sequence. As defined in a roundtable consensus report [7], each of four quadrants is assigned a score (0 = no flow, 1 = intermittent flow, 2 = sluggish, 3 = continuous) based on the predominant flow in the vessels (i.e., the statistical mode) which occupy that quadrant. The MFI score is the average of the predominant flow score over the quadrants. To compute the microcirculatory parameters TVD, PVD, and PPV for small, medium, and large vessels, the vessels are classified automatically by their average width. Vessel lengths are summed for each size category to compute total vessel length, *L*_v_ *.* Perfused vessel lengths are summed for each size category to compute perfused vessel length, *L*_p_. Most analyses of microcirculatory flow are restricted to vessels 0–20 μm because these are the most physiologically active vessels for oxygen exchange. Table 2 presents the symbols and formulas of the parameters with dimensional units.

## Section 5: conclusion

### Ongoing challenges

Higher quality and/or lower cost SDF/IDF cameras are becoming available for research use. Even as the technology becomes more accessible, image quality and analysis challenges remain significant hurdles. Data loss and missing data because of poor quality are a costly use of clinician and analysis resources. A large amount of raw data typically must be discarded because of image quality artifacts, mainly due to a poor acquisition technique. Manually tracing vessels is too time consuming to be practical for clinical utility. Clinicians require automated methods that provide data for actionable results. Manufacturers and researchers are developing promising new algorithms for automated analysis, but there is no universally accepted solution currently available.

### Future work

We expect that consumer imaging technologies will drive down the price of SDF/IDF cameras significantly. Thus, access to these cameras could become widespread. Smartphone apps that integrate multiple sensor data with sublingual microvascular imaging could allow onsite or remote analysis for battlefield, telemedicine, or in-home patient monitoring and diagnoses. However, automated capture and analysis software and actionable image-based metrics have been disappointing to date. Furthermore, acquisition artifacts, especially pressure artifacts, remain an important hurdle. We intend to apply our manual analysis technique as a gold standard for establishing ground-truth datasets for testing and training automatic vessel detection and image quality assessment algorithms.

### Conclusion

This review has provided a summary of sublingual microcirculatory image capture and analysis. We briefly reviewed several imaging technologies and their accompanying software applications that have been used to date. We summarized and proposed best practices to mitigate image quality artifacts during image acquisition. We outlined video data management using a relational database and network storage. We walked the reader through the image analysis pipeline, discussing vessel segmentation, tracing, velocity scoring, and extracting microcirculation parameters. Finally, we suggest that addressing improved usability and automated analysis should be avenues of continued device development.

## Additional files

Additional file 1: **Microsoft Word document presenting ParseReports.bas, a Visual Basic script to be executed in Microsoft Access that parses AVA 3.1 Report files to extract relevant microcirculatory parameters and metadata.** (DOCX 20 kb)

Additional file 2: **Microsoft Word document presenting a summary of microvascular semiquantitative flow characterization.** These categories were originally defined to describe microcirculatory flow in sepsis pathophysiology. (DOCX 13 kb)

## Abbreviations

DV: Digital video
ED: Emergency department
IDF: Incident dark field
IQS: Image quality score
JPEG: Joint photographic experts group
LED: Light-emitting diode
MFI: Microvascular flow index
M-JPEG: Motion joint photographic experts group
MP4: Moving pictures expert group-4, video compression codec
MPEG: Moving pictures expert group
NTSC: National television system committee
OPS: Orthogonal polarization spectral
OPS-DF: Orthogonal polarization spectral dark field
PAL: Phase alternating line
PPV: Proportion of perfused vessels
PVD: Perfused vessel density
RBC: Red blood cell
SDF: Sidestream dark field
TVD: Total vessel density
UI: User interface

## References

[1] Groner W, Winkelman JW, Harris AG, Ince C, Bouma GJ, Messmer K (1999). Orthogonal polarization spectral imaging: a new method for study of the microcirculation. Nat Med.

[2] Arnold RC, Parrillo JE, Phillip Dellinger R, Chansky ME, Shapiro NI, Lundy DJ (2009). Point-of-care assessment of microvascular blood flow in critically ill patients. Intensive Care Med.

[3] Bezemer R, Bartels SA, Bakker J, Ince C (2012). Clinical review: Clinical imaging of the sublingual microcirculation in the critically ill—where do we stand. Crit Care.

[4] Bezemer R, Dobbe JG, Bartels SA, Boerma EC, Elbers PW, Heger M (2011). Rapid automatic assessment of microvascular density in sidestream dark field images. Med Biol Eng Comput.

[5] Boerma EC, Mathura KR, van der Voort PH, Spronk PE, Ince C (2005). Quantifying bedside-derived imaging of microcirculatory abnormalities in septic patients: a prospective validation study. Crit Care.

[6] Bracht H, Krejci V, Hiltebrand L, Brandt S, Sigurdsson G, Ali SZ (2008). Orthogonal polarization spectroscopy to detect mesenteric hypoperfusion. Intensive Care Med.

[7] De Backer D, Hollenberg S, Boerma C, Goedhart P, Buchele G, Ospina-Tascon G (2007). How to evaluate the microcirculation: report of a round table conference. Crit Care.

[8] De Backer D, Ospina-Tascon G, Salgado D, Favory R, Creteur J, Vincent JL (2010). Monitoring the microcirculation in the critically ill patient: current methods and future approaches. Intensive Care Med.

[9] De Backer D, Verdant C, Chierego M, Koch M, Gullo A, Vincent JL (2006). Effects of drotrecogin alfa activated on microcirculatory alterations in patients with severe sepsis. Crit Care Med.

[10] den Uil CA, Caliskan K, Lagrand WK, van der Ent M, Jewbali LS, van Kuijk JP (2009). Dose-dependent benefit of nitroglycerin on microcirculation of patients with severe heart failure. Intensive Care Med.

[11] den Uil CA, Lagrand WK, Spronk PE, van der Ent M, Jewbali LS, Brugts JJ (2009). Low-dose nitroglycerin improves microcirculation in hospitalized patients with acute heart failure. Eur J Heart Fail.

[12] Dobbe JG, Streekstra GJ, Atasever B, van Zijderveld R, Ince C (2008). Measurement of functional microcirculatory geometry and velocity distributions using automated image analysis. Med Biol Eng Comput.

[13] Donati A, Damiani E, Domizi R, Romano R, Adrario E, Pelaia P (2013). Alteration of the sublingual microvascular glycocalyx in critically ill patients. Microvasc Res.

[14] Donati A, Domizi R, Damiani E, Adrario E, Pelaia P, Ince C (2013). From macrohemodynamic to the microcirculation. Crit Care Res Pract.

[15] Kanoore Edul VS, Enrico C, Laviolle B, Vazquez AR, Ince C, Dubin A (2012). Quantitative assessment of the microcirculation in healthy volunteers and in patients with septic shock. Crit Care Med.

[16] Elbers PW, Ince C (2006). Mechanisms of critical illness—classifying microcirculatory flow abnormalities in distributive shock. Crit Care.

[17] Ellis CG, Jagger J, Sharpe M (2005). The microcirculation as a functional system. Crit Care.

[18] Eriksson S, Nilsson J, Sturesson C (2014). Non-invasive imaging of microcirculation: a technology review. Med Devices (Auckl).

[19] Ince C (2008). The elusive microcirculation. Intensive Care Med.

[20] Ince C (2005). The microcirculation is the motor of sepsis. Crit Care.

[21] Massey MJ, Larochelle E, Najarro G, Karmacharla A, Arnold R, Trzeciak S (2013). The microcirculation image quality score: development and preliminary evaluation of a proposed approach to grading quality of image acquisition for bedside videomicroscopy. J Crit Care.

[22] Milstein DMJ, Lindeboom JAH, Ince C. Sidestream dark-field imaging and image analysis of oral microcirculation under clinical conditions. Anaesth Pain Intensive Care Emerg. Proceedings of the 20th Postgraduate Course in Critical Care Medicine Trieste, Italy—November 18–21, 2005. 2006;Chapter 6:79–88.

[23] Omar YG, Massey M, Andersen LW, Giberson TA, Berg K, Cocchi MN (2013). Sublingual microcirculation is impaired in post-cardiac arrest patients. Resuscitation.

[24] Robertson R, Lockhart E, Shapiro NI, Bankarenko N, McMahon T, Massey MJ (2012). Impact of transfusion of autologous 7 versus 42 day old AS-3 red blood cells on tissue oxygenation and the microcirculation in healthy volunteers. Transfusion.

[25] Sakr Y, Chierego M, Piagnerelli M, Verdant C, Dubois MJ, Koch M (2007). Microvascular response to red blood cell transfusion in patients with severe sepsis. Crit Care Med.

[26] Sallisalmi M. Microcirculation and hemorheology in critically ill patients. PhD thesis, Dept. of Surgery Division of Anesthesia and Intensive Care Medicine. University of Helsinki, Helsinki, Finland. 2013. ISBN 978-952-10-8687-8 (PDF).

[27] Sallisalmi M, Oksala N, Pettila V, Tenhunen J (2012). Evaluation of sublingual microcirculatory blood flow in the critically ill. Acta Anaesthesiol Scand.

[28] Sheikh MY, Javed U, Singh J, Choudhury J, Deen O, Dhah K (2009). Bedside sublingual video imaging of microcirculation in assessing bacterial infection in cirrhosis. Dig Dis Sci.

[29] Struijker-Boudier HA, Rosei AE, Bruneval P, Camici PG, Christ F, Henrion D (2007). Evaluation of the microcirculation in hypertension and cardiovascular disease. Eur Heart J.

[30] Taccone FS, Su F, Pierrakos C, He X, James S, Dewitte O (2010). Cerebral microcirculation is impaired during sepsis: an experimental study. Crit Care.

[31] Trzeciak S, Dellinger RP, Parrillo JE, Guglielmi M, Bajaj J, Abate NL (2007). Early microcirculatory perfusion derangements in patients with severe sepsis and septic shock: relationship to hemodynamics, oxygen transport, and survival. Ann Emerg Med.

[32] Trzeciak S, McCoy JV, Phillip Dellinger R, Arnold RC, Rizzuto M, Abate NL (2008). Early increases in microcirculatory perfusion during protocol-directed resuscitation are associated with reduced multi-organ failure at 24 h in patients with sepsis. Intensive Care Med.

[33] Trzeciak S, Rivers EP (2005). Clinical manifestations of disordered microcirculatory perfusion in severe sepsis. Crit Care.

[34] van Beers EJ, Goedhart PT, Unger M, Biemond BJ, Ince C (2008). Normal sublingual microcirculation during painful crisis in sickle cell disease. Microvasc Res.

[35] van Elteren HA, Ince C, Tibboel D, Reiss IK, de Jonge RC (2015). Cutaneous microcirculation in preterm neonates: comparison between sidestream dark field (SDF) and incident dark field (IDF) imaging. J Clin Monit Comput.

[36] Verdant CL, De Backer D, Bruhn A, Clausi CM, Su F, Wang Z (2009). Evaluation of sublingual and gut mucosal microcirculation in sepsis: a quantitative analysis. Crit Care Med.

[37] Vincent JL, De Backer D (2005). Microvascular dysfunction as a cause of organ dysfunction in severe sepsis. Crit Care.

[38] Vellinga NA, Boerma EC, Koopmans M, Donati A, Dubin A, Shapiro NI (2015). International study on microcirculatory shock occurrence in acutely ill patients. Crit Care Med.

[39] Dababneh L, Cikach F, Alkukhun L, Dweik RA, Tonelli AR (2014). Sublingual microcirculation in pulmonary arterial hypertension. Ann Am Thorac Soc.

[40] Aykut G, Veenstra G, Scorcella C, Ince C, Boerma C (2015). Cytocam-IDF (incident dark field illumination) imaging for bedside monitoring of the microcirculation. Intensive Care Med Exp.

[41] Bezemer R, Khalilzada M, Ince C (2008). Recent advancements in microcirculatory image acquisition and analysis. Yearbook of intensive care and emergency medicine.

[42] De Backer D, Creteur J, Preiser JC, Dubois MJ, Vincent JL (2002). Microvascular blood flow is altered in patients with sepsis. Am J Respir Crit Care Med.

[43] Sakr Y, Dubois MJ, De Backer D, Creteur J, Vincent JL (2004). Persistent microcirculatory alterations are associated with organ failure and death in patients with septic shock. Crit Care Med.

[44] Spronk PE, Ince C, Gardien MJ, Mathura KR, Oudemans-van Straaten HM, Zandstra DF (2002). Nitroglycerin in septic shock after intravascular volume resuscitation. Lancet.

[45] Sherman H, Klausner S, Cook WA (1971). Incident dark-field illumination a new method for microcirculation study. Angiology.

[46] Goedhart PT, Khalilzada M, Bezemer R, Merza J, Ince C (2007). Sidestream dark field (SDF) imaging: a novel stroboscopic LED ring-based imaging modality for clinical assessment of the microcirculation. Opt Express.

[47] Balestra GM, Bezemer R, Boerma EC, Yong ZY, Sjauw KD, Engstrom AE (2010). Improvement of sidestream dark field imaging with an image acquisition stabilizer. BMC Med Imaging.

[48] Lindert J, Werner J, Redlin M, Kuppe H, Habazettl H, Pries AR (2002). OPS imaging of human microcirculation. A short technical report. J Vasc Res.

[49] Demir SU, Mirshahi N, Tiba MH, Draucker G, Ward KR, Hobson R, et al. Image processing and machine learning for diagnostic analysis of microcirculation. ICME International Conference Complex Medical Engineering, 9–11 April 2009. 2009:1–5. http://ieeexplore.ieee.org/xpl/abstractSimilar.jsp?tp=&arnumber=4906669&url=http%3A%2F%2Fieeexplore.ieee.org%2Fxpls%2Fabs_all.jsp%3Farnumber%3D4906669.

[50] Demir SU, Hakimzadeh R, Hargraves RH, Ward KR, Myer EV, Najarian K (2012). An automated method for analysis of microcirculation videos for accurate assessment of tissue perfusion. BMC Med Imaging.

[51] Demir SU, Mirshahi N. Ward K, Hakimzadeh R. Hobson R, Najarian K. Vessel extraction of microcirculatory video recordings using multi-thresholding based verification algorithm. 2010 International Conference on Biosciences (BIOSCIENCESWORLD); 7–13 March 2010:11–15. doi:10.1109/BioSciencesWorld.2010.9

[52] You S, Ataer-Cansizoglu E, Erdogmus D, Massey M, Shapiro N. Microvascular blood flow estimation in sublingual microcirculation videos based on a principal curve tracing algorithm. 2012 IEEE Int Workshop Machine Learning for Signal Proc (MLSP); 23–26 Sept. 2012:1–6. doi:10.1109/MLSP.2012.6349763

[53] You S, Massey M, Shapiro N, Erdogmus D. A novel line detection method in space-time images for microvascular blood flow analysis in sublingual microcirculatory videos. 2013 IEEE 10th Int Symposium on Biomed Imaging; 7–11 April 2013:828–31. doi:10.1109/ISBI.2013.6556603.

[54] Pozo MO, Kanoore Edul VS, Ince C, Dubin A (2012). Comparison of different methods for the calculation of the microvascular flow index. Crit Care Res Pract.

