# Supplementary material for: A guide to human in vivo microcirculatory flow image analysis
Source: Crit Care. 2016 Feb 10;20:35. doi: 10.1186/s13054-016-1213-9 (PMC4748457; doi:10.1186/s13054-016-1213-9)
Supplement: Supplementary file 1 — Microsoft Word document presenting ParseReports.bas, a Visual Basic script to be executed in Microsoft Access that parses AVA 3.1 Report files to extract relevant microcirculatory parameters and metadata. (DOCX 20 kb) [file 13054_2016_1213_MOESM1_ESM.docx]

Additional file 1: ParseReports.bas

Below is a Visual Basic script that uses regular expressions to parse an AVA report file and extract parametric data into a table.

Attribute VB_Name = "ParseReports"

Option Compare Database

Option Explicit

Private Const FILE_EXT = ".txt"

Private Const sQ = "'"

Private sQuery As String

Private Const sResultsTable = "tblAnalysisResults"

Private sUpdateCommand As String

Private sFieldList As String

Private sValueList As String

Private sSqlWhere As String

Private sUpdateList As String

Public Sub AppendUpdateValue(sField As String, sValue As Variant)

If sFieldList = "" And sValueList = "" Then

sFieldList = sField

If Not IsNumeric(sValue) Then

sValueList = sQ + sValue + sQ

sUpdateList = sField + " = " + sQ + sValue + sQ

Else

sValueList = sValue

sUpdateList = sField + " = " + sValue

End If

Else

sFieldList = sFieldList + "," + sField

If Not IsNumeric(sValue) Then

sValueList = sValueList + "," + sQ + sValue + sQ

sUpdateList = sUpdateList + ", " + sField + " = " + sQ + sValue + sQ

Else

sValueList = sValueList + "," + CStr(sValue)

sUpdateList = sUpdateList + ", " + sField + " = " + CStr(sValue)

End If

End If

End Sub

Public Sub InitUpdateCommand()

sUpdateCommand = "UPDATE " + sResultsTable + " SET "

sValueList = ""

sFieldList = ""

sSqlWhere = ""

sUpdateList = ""

End Sub

Public Function GetUpdateCommand() As String

Dim sCmd As String

sCmd = sUpdateCommand + sUpdateList + sSqlWhere

GetUpdateCommand = sCmd

End Function

Public Sub ParseReportFast(curFile As String)

Dim lFile As Long

Dim szLine As String, tmpPattern As String

Dim regex As New RegExp

Dim colregmatch As MatchCollection

Dim subs As submatches

Dim regmatch As match

Dim vfname As String

Dim vfparts As Variant

Dim vfname2 As String

Dim q As String

Dim sqlWhere As String

Dim sRandID As String

Dim sQueryStr As String

Dim vSize As String

Dim bVesselDensity As Boolean

Dim bVesselFlow As Boolean

Dim bBoermaVelocity As Boolean

Dim ShapiroPPV, ShapiroPPV_1, ShapiroPPV_2, ShapiroPPV_3 As Single

Dim sDate As String

Dim rs As DAO.Recordset

Dim LogbookDate As Date

Dim VLength As Single

Dim PerfVLength As Single

Dim fSqv0 As Single

Dim fSqv1 As Single

Dim fSqv2 As Single

Dim fSqv3 As Single

Dim fSqvTotal As Single

With regex

.MultiLine = False

.Global = True

.IgnoreCase = True

End With

lFile = FreeFile()

Open curFile For Input As lFile

While Not EOF(lFile)

Line Input #lFile, szLine

'file name: so you can correlate it in the database:

regex.Pattern = "^Video file"

If regex.Test(szLine) Then

regex.Pattern = "^Video file.*?\)[\t ]+(.*?)$"

Set colregmatch = regex.Execute(szLine)

vfname = colregmatch.Item(0).submatches.Item(0)

vfname = Replace(vfname, "\", "&&")

vfparts = Split(vfname, "&&")

vfname2 = vfparts(UBound(vfparts))

vfparts = Split(vfname2, "_")

sRandID = vfparts(0)

If Not isRecordInDB(sRandID, "RandomizedID", sResultsTable, rs) Then

q = "INSERT INTO " + sResultsTable + " (RandomizedID,VideoFileName) _

VALUES ('" + vfparts(0) + "','" + vfname2 + "')"

Application.CurrentDb.Execute (q)

ElseIf Not IsNull(rs("LogbookDate")) Then

LogbookDate = rs("LogbookDate")

End If

InitUpdateCommand

AppendUpdateValue "VideoFileName", vfname2

sqlWhere = " WHERE (RandomizedID='" + vfparts(0) + "') " '+ _

"AND (VideoFileName='" + vfname2 + "')"

sSqlWhere = sqlWhere

End If

'Logbook file date:

regex.Pattern = "^Logbook file stored.*?y-m-d.*?"

If regex.Test(szLine) Then

regex.Pattern = _

".*?([0-9]+)-([0-9][0-9])-([0-9][0-9]).*?([0-9][0-9]):([0-9][0-9]):([0-9][0-9]).*?"

Set colregmatch = regex.Execute(szLine)

sDate = colregmatch.Item(0).submatches.Item(0) + "/" + _

colregmatch.Item(0).submatches.Item(1) + "/" + _

colregmatch.Item(0).submatches.Item(2) + " " + _

colregmatch.Item(0).submatches.Item(3) + ":" + _

colregmatch.Item(0).submatches.Item(4) + ":" + _

colregmatch.Item(0).submatches.Item(5)

If CDate(sDate) <= LogbookDate Then

Close lFile

Exit Sub

End If

AppendUpdateValue "LogbookDate", sDate

End If

'Area of ROI:

regex.Pattern = "^Area of ROI.*?um2.*?"

If regex.Test(szLine) Then

regex.Pattern = ".*?um2.*?([0-9]+)"

Set colregmatch = regex.Execute(szLine)

AppendUpdateValue "AreaROI", colregmatch.Item(0).submatches.Item(0)

End If

'horizontal pitch:

'horPitch

regex.Pattern = "^Horizontal pitch"

If regex.Test(szLine) Then

regex.Pattern = ".*?([0-9.]+)"

Set colregmatch = regex.Execute(szLine)

AppendUpdateValue "horPitch", colregmatch.Item(0).submatches.Item(0)

End If

'Vertical pitch:

'vertPitch

regex.Pattern = "^Vertical pitch"

If regex.Test(szLine) Then

regex.Pattern = ".*?([0-9.]+)"

Set colregmatch = regex.Execute(szLine)

AppendUpdateValue "vertPitch", colregmatch.Item(0).submatches.Item(0)

End If

'detected vessel length:

'vesselLength

regex.Pattern = "^Detected vessel length.*?mm.*?"

If regex.Test(szLine) Then

regex.Pattern = ".*?([0-9.]+)"

Set colregmatch = regex.Execute(szLine)

AppendUpdateValue "vesselLength", colregmatch.Item(0).submatches.Item(0)

End If

'Vessel density:

regex.Pattern = "^Vessel density.*?mm.*?mm2"

If regex.Test(szLine) Then

regex.Pattern = ".*?mm.*?mm2.*?([0-9.]+)"

Set colregmatch = regex.Execute(szLine)

AppendUpdateValue "vesselDensity", colregmatch.Item(0).submatches.Item(0)

End If

'Vessel surface:

regex.Pattern = "^Vessel surface.*?mm.*?mm2"

If regex.Test(szLine) Then

regex.Pattern = ".*?mm.*?mm2.*?100.*?([0-9.]+)"

Set colregmatch = regex.Execute(szLine)

AppendUpdateValue "vesselSurface", colregmatch.Item(0).submatches.Item(0)

End If

'De Backer score:

regex.Pattern = "^De Backer score"

If regex.Test(szLine) Then

regex.Pattern = ".*?mm.*?([0-9.]+)"

Set colregmatch = regex.Execute(szLine)

AppendUpdateValue "DeBackerScore", colregmatch.Item(0).submatches.Item(0)

End If

regex.Pattern = "^Semi-quantitative velocity scoring.*?total vessel length"

If regex.Test(szLine) Then

bVesselFlow = True

End If

If bVesselFlow Then

'Small 0.0..20.0 0.00 1.49 25.71 41.05 12.31 0.00 80.56

'Grab the last 6 #s into separate groups

tmpPattern = _

"^(Small|Medium).*?[0-9.]+.*?[0-9.]+.*?([0-9.]+).*?([0-9.]+).*?([0-9.]+).*?([0-9.]+).*?([0-9.]+).*?([0-9.]+)"

regex.Pattern = "^(Small|Medium|Large)"

If regex.Test(szLine) Then

regex.Pattern = tmpPattern

Set colregmatch = regex.Execute(szLine)

vSize = colregmatch.Item(0).submatches.Item(0)

fSqv0 = colregmatch.Item(0).submatches.Item(1)

fSqv1 = colregmatch.Item(0).submatches.Item(2)

fSqv2 = colregmatch.Item(0).submatches.Item(3)

fSqv3 = colregmatch.Item(0).submatches.Item(4)

fSqvTotal = colregmatch.Item(0).submatches.Item(6)

AppendUpdateValue "sqv" + vSize + "NoFlow", CStr(fSqv0)

AppendUpdateValue "sqv" + vSize + "Intermittent", CStr(fSqv1)

AppendUpdateValue "sqv" + vSize + "Sluggish", CStr(fSqv2)

AppendUpdateValue "sqv" + vSize + "Continuous", CStr(fSqv3)

AppendUpdateValue "sqv" + vSize + "Total", CStr(fSqvTotal)

If fSqvTotal > 0 Then

ShapiroPPV_1 = (fSqv1 + fSqv2 + fSqv3) / fSqvTotal

ShapiroPPV_2 = (fSqv2 + fSqv3) / fSqvTotal

ShapiroPPV_3 = fSqv3 / fSqvTotal

End If

AppendUpdateValue "ShapiroPPV1" + vSize, CStr(ShapiroPPV_1)

AppendUpdateValue "ShapiroPPV2" + vSize, CStr(ShapiroPPV_2)

AppendUpdateValue "ShapiroPPV3" + vSize, CStr(ShapiroPPV_3)

AppendUpdateValue "ShapiroPPV_" + vSize, CStr(ShapiroPPV_2)

'Stop dealing with the vessel density block after we've reached "Medium"

bVesselFlow = Not colregmatch.Item(0).submatches.Item(0) = "Medium"

End If

End If

regex.Pattern = "^Boerma velocity classification"

If regex.Test(szLine) Then

bBoermaVelocity = True

End If

If bBoermaVelocity Then

'Large 50.0..100.0 3 3 - - 3.0

'Grab the last 6 #s into separate groups

tmpPattern = _

"^(Small|Medium).*?[0-9.]+.*?([0-9.-]+).*?([0-9.-]+).*?([0-9.-]+).*?([0-9.-]+).*?([0-9.-]+)"

regex.Pattern = "^(Small|Medium)"

If regex.Test(szLine) Then

regex.Pattern = tmpPattern

Set colregmatch = regex.Execute(szLine)

vSize = colregmatch.Item(0).submatches.Item(0)

AppendUpdateValue "mfi" + vSize + "Q1", _

colregmatch.Item(0).submatches.Item(1)

AppendUpdateValue "mfi" + vSize + "Q2", _

colregmatch.Item(0).submatches.Item(2)

AppendUpdateValue "mfi" + vSize + "Q3", _

colregmatch.Item(0).submatches.Item(3)

AppendUpdateValue "mfi" + vSize + "Q4", _

colregmatch.Item(0).submatches.Item(4)

'mmassey 2012-12-11 compute average mfi to account for missing values:

Dim mfival As Double, mficount As Double, mfiall As Double

mficount = 0

mfival = 0

mfiall = -99

If Not colregmatch.Item(0).submatches.Item(1) = "-" Then

If val(colregmatch.Item(0).submatches.Item(1)) >= 0 And _

val(colregmatch.Item(0).submatches.Item(1)) < 4 Then

mficount = mficount + 1

mfival = mfival + val(colregmatch.Item(0).submatches.Item(1))

End If

Else

Debug.Print mficount; mfival

End If

If Not colregmatch.Item(0).submatches.Item(2) = "-" Then

If val(colregmatch.Item(0).submatches.Item(2)) >= 0 And _

val(colregmatch.Item(0).submatches.Item(2)) < 4 Then

mficount = mficount + 1

mfival = mfival + val(colregmatch.Item(0).submatches.Item(2))

End If

Else

Debug.Print mficount; mfival

End If

If Not colregmatch.Item(0).submatches.Item(3) = "-" Then

If val(colregmatch.Item(0).submatches.Item(3)) >= 0 And _

val(colregmatch.Item(0).submatches.Item(3)) < 4 Then

mficount = mficount + 1

mfival = mfival + val(colregmatch.Item(0).submatches.Item(3))

End If

Else

Debug.Print mficount; mfival

End If

If Not colregmatch.Item(0).submatches.Item(4) = "-" Then

If val(colregmatch.Item(0).submatches.Item(4)) >= 0 And _

val(colregmatch.Item(0).submatches.Item(4)) < 4 Then

mficount = mficount + 1

mfival = mfival + val(colregmatch.Item(0).submatches.Item(4))

End If

Else

Debug.Print mficount; mfival

End If

If mficount > 0 Then

mfiall = mfival / mficount

Else

mfiall = -99

End If

AppendUpdateValue "mfi" + vSize + "All", mfiall

'Stop dealing with the Boerma velocity block after we've reached "Medium"

bBoermaVelocity = _

Not colregmatch.Item(0).submatches.Item(0) = "Medium"

End If

End If

'Extract Report Summary information:TVD

regex.Pattern = "^Total Vessel Density"

If regex.Test(szLine) Then

regex.Pattern = ".*?mm.*?mm2.*?([0-9.]+).*?([0-9.-]+).*?([0-9.-]+)"

Set colregmatch = regex.Execute(szLine)

AppendUpdateValue "SummaryTVD_Small", _

colregmatch.Item(0).submatches.Item(0)

AppendUpdateValue "SummaryTVD_Other", _

colregmatch.Item(0).submatches.Item(1)

AppendUpdateValue "SummaryTVD_All", _

colregmatch.Item(0).submatches.Item(2)

End If

'Extract Report Summary information:PVD

regex.Pattern = "^Perfused Vessel Density"

If regex.Test(szLine) Then

regex.Pattern = ".*?mm.*?mm2.*?([0-9.]+).*?([0-9.-]+).*?([0-9.-]+)"

Set colregmatch = regex.Execute(szLine)

AppendUpdateValue "SummaryPVD_Small", _

colregmatch.Item(0).submatches.Item(0)

AppendUpdateValue "SummaryPVD_Other", _

colregmatch.Item(0).submatches.Item(1)

AppendUpdateValue "SummaryPVD_All", _

colregmatch.Item(0).submatches.Item(2)

End If

'Extract Report Summary information:PPV

regex.Pattern = "^Proportion of Perfused vessels"

If regex.Test(szLine) Then

regex.Pattern = ".*?.*?([0-9.]+).*?([0-9.-]+).*?([0-9.-]+)"

Set colregmatch = regex.Execute(szLine)

AppendUpdateValue "SummaryPPV_Small", _

colregmatch.Item(0).submatches.Item(0)

AppendUpdateValue "SummaryPPV_Other", _

colregmatch.Item(0).submatches.Item(1)

AppendUpdateValue "SummaryPPV_All", _

colregmatch.Item(0).submatches.Item(2)

End If

Wend

AppendUpdateValue "Path", curFile

Dim strArray() As String

strArray = Split(curFile, "\")

AppendUpdateValue "ReportName", strArray(UBound(strArray))

AppendUpdateValue "Date_Modified", CStr(Now)

sQueryStr = GetUpdateCommand

Debug.Print sQueryStr

Application.CurrentDb.Execute (sQueryStr)

'Set AnalysisComplete flag to yes

sQueryStr = "UPDATE tblClippedVideoFiles SET AnalysisComplete = '-1' _

WHERE RandomizedID = '" + sRandID + "'"

Application.CurrentDb.Execute (sQueryStr)

Close lFile

End Sub

Private Function isRecordInDB(sValue As String, sField As String, sTable As String, rs As DAO.Recordset) As Boolean

Dim Query As String

'Dim rs As dao.Recordset

Query = "SELECT * FROM " + sTable + " WHERE " + sField + "='" + sValue + sQ

Set rs = Application.CurrentDb.OpenRecordset(Query)

isRecordInDB = Not (rs.EOF And rs.BOF)

End Function
