# Supplementary material for: A guide to human in vivo microcirculatory flow image analysis
Source: Crit Care. 2016 Feb 10;20:35. doi: 10.1186/s13054-016-1213-9 (PMC4748457; doi:10.1186/s13054-016-1213-9)
Supplement: Supplementary file 2 — Microsoft Word document presenting a summary of microvascular semiquantitative flow characterization. These categories were originally defined to describe microcirculatory flow in sepsis pathophysiology. (DOCX 13 kb) [file 13054_2016_1213_MOESM2_ESM.docx]

Microvascular Semi-Quantitative Flow Characterization:

Below is a summary of semi-quantitative flow classification. These categories were originally defined to describe microcirculatory flow in sepsis pathophysiology.

Continuous Flow:

Steady, unimpeded stream of RBCs; Or a continuous train of RBCs and plasma gaps in a capillary. Typically speed > 300 um/s.

Sluggish Flow:

A continuous stream of RBCs with speed approximately 80 – 300 um/s; Or a non-constant flow with minimum speed between approximately 80 – 150 um/s.

Intermittent Flow:

Very slowly moving or non-continuous stream of RBCs in a vessel. Individual RBCs may be fast moving but infrequent or very slowly moving flow approximately < 80 um/s such that some RBCs clear a vessel during the scan duration; Or flow that slows, stops and reverses direction, but some RBCs clear the vessel.

No Flow:

RBCs do not clear the vessel throughout the duration of the scan.
